# Evolution and Regulation of Radial Structure of PAN Pre-Oxidized Fiber Based on the Fine Denier Model

**DOI:** 10.3390/ma15041409

**Published:** 2022-02-14

**Authors:** Bin Wang, Yunfeng Wang, Chenggao Li, Aijun Gao

**Affiliations:** 1 Central Research Institute of Building and Construction Co., Ltd., MCC, Beijing 100088, China; mccwangbin@126.com; 2 Key Laboratory of Carbon Fiber and Functional Polymers of the Ministry of Education, Beijing University of Chemical Technology, Beijing 100029, China; 18811356065@163.com; 3 Key Lab of Structures Dynamic Behavior and Control of the Ministry of Education, Harbin Institute of Technology, Harbin 150090, China; 4 Key Lab of Smart Prevention and Mitigation of Civil Engineering Disasters of the Ministry of Industry and Information Technology, Harbin Institute of Technology, Harbin 150090, China; 5 School of Civil Engineering, Harbin Institute of Technology, Harbin 150090, China

**Keywords:** radial structure, PAN pre-oxidized fiber, evolution mechanism, microstructure analysis, carbon fiber

## Abstract

Affected by ambient oxygen and thermal diffusion mechanism, the radial structural distribution of polyacrylonitrile (PAN) fiber during the pre-oxidation process will be inherited to the carbon fiber, which had a remarkable effect on the mechanical properties of carbon fiber. It is important to understand the evolution mechanism of radial structure evolution of PAN fiber during the pre-oxidation process to manufacture the high-performance carbon fiber. In this paper, a series of fine denier model fibers were prepared by adjusting the oxygen concentration to describe the structural characteristics at different radial regions of pre-oxidized fibers. The evolution mechanism of the radial structure of pre-oxidized fiber, with the increase of heat treatment temperature, was studied by the methods of optical microscope, C^13^ nuclear magnetic resonance (^13^C-NMR), and thermogravimetric analyzer (TGA). The results showed that along the radial direction of pre-oxidized fiber from skin to core layer, the degree of cyclization changed little while the dehydrogenation and oxygen-containing structure gradually decreased. Specifically, the oxygen-containing functional groups in the core decreased to the lowest level or even disappeared. A moderate increase of temperature in the initial and middle pre-oxidation processes could effectively promote the formation of cyclized structure and stabilize cross-linked ladder structure in the skin region of the fiber. With it, the thermal stability of obtained pre-oxidation fiber was improved.

## 1. Introduction

Polyacrylonitrile (PAN) based carbon fiber has excellent mechanical and functional properties, which made it an irreplaceable material in aerospace, energy transportation, civil and construction engineering, bridge structures, and other fields [1,2,3,4]. The preparation technology of high-performance carbon fiber was always the main task for the researchers. How to control the structural homogeneity is still one of the key problems to be solved [5,6,7].

**Figure 1:**
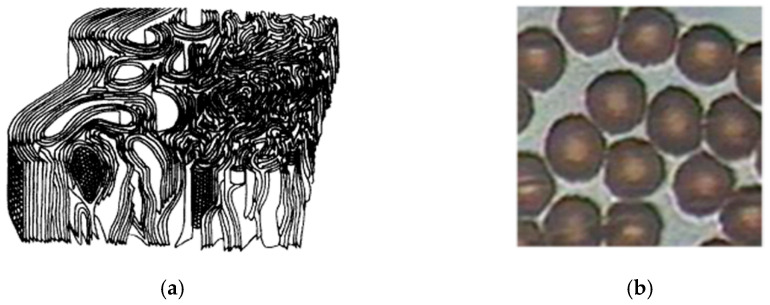
Skin-core structure of PAN fiber of (**a**) skin-core structural model of carbon fiber [10] and (**b**) micrograph of skin-core structure of pre-oxidized fiber [12].

Compared with the ideal graphite, PAN-based carbon fiber has a “pseudo-graphite” structure with a poor three-dimensional order and a large number of defects [8,9]. In 1974, Johnson et al. [10,11] proposed the skin-core structure model of PAN-based carbon fiber, as shown in Figure 1, including the skin structure and core structure. Furthermore, the arrangement of a “pseudo-graphite” structure was relatively regular in the skin, while it was arranged disorderly in the core.

In the past decades, it was found that PAN-based pre-oxidized fiber presented on a unique radial structure because of ambient oxygen diffusion kinetics and thermal transferring mechanism. This difference in radial structure would further affect the development of skin-core structure of the fiber during the carbonization process. This played a key role in the mechanical properties of ultimate carbon fiber [12,13,14].

Xue et al. [15,16] reported that the pre-oxidation degree of PAN-based pre-oxidized fiber in the skin was higher, while it was the lowest in the core due to the decrease of oxygen content. The skin-core structure of pre-oxidized fibers could be weakened through the thermal treatment at a lower temperature for a long time, according to the work [17,18]. However, it was also easy to form the excessive oxygenated functional groups in the fiber. In the subsequent carbonization process, more oxygenated functional groups were cracked and removed in the form of small molecules, resulting in a large number of pore defects of carbon fiber. This was detrimental to the improvement of the mechanical properties of carbon fiber. Some studies showed that the formation of the skin-core structure of PAN-based pre-oxidized fiber was dependent on the heat treatment temperature. When the temperature was 210–240 °C, the skin-core structure of fiber was not obvious while the pre-oxidation reaction was intense. Furthermore, the skin-core ratio of fiber increased rapidly when the temperature was increased to 240–260 °C. It was proposed that the skin-core structure could be applied to characterize the degree of pre-oxidation reaction. It was even proposed that the tensile strength of PAN-based carbon fiber increased first and then decreased with the increase of pre-oxidation degree [19,20]. For conventional PAN-based pre-oxidized fiber, the fiber diameter was usually about 10~15 μm, which made it difficult for the available characterization methods to distinguish the difference between the skin and core region. So far, the effect mechanisms of oxygen concentration and heat treatment temperature on the chemical structure along the fiber radial direction during the pre-oxidation process have not been well clarified.

In this paper, a fine denier model fiber was prepared in the laboratory, which was beneficial to form more homogeneous pre-oxidation structures owing to the full diffusion of ambient oxygen into the fiber. To simulate the radial structure of fiber during the pre-oxidation process, a series of fine denier pre-oxidized fiber samples corresponding to different radial structures were prepared by changing the relative content of oxygen. By the methods of optical microscope, ^13^C-NMR, and TGA, the chemical characteristics of the radial structure of pre-oxidized fiber and its evolution mechanism with the increase of temperature were studied. Moreover, the conventional-size fibers were used to reveal the effects of temperature at different stages on the evolution of fiber radial structures and the formation of thermally stabilized structures. Thus, the radial structure distribution of fiber during the pre-oxidation process was optimized, which provided an effective method for the preparation of high-performance carbon fiber.

## 2. Materials and Methods

### 2.1. Raw Materials and Sample Preparation

The preparation process of PAN precursor fibers was as follows: The wet spinning process was applied to produce the precursor fibers with different diameters in the laboratory. The precursor was composed of acrylonitrile and itaconic acid copolymer, and the content of itaconic acid was 0.5%. Two kinds of precursors were obtained. Precursor A (fine denier) included 1000 filaments per tow, and the diameter of each filament was about 9 μm. Precursor B (conventional size) included 1000 filaments per tow, and the diameter of each filament was about 15 μm.

The preparation process of PAN-based pre-oxidized fibers was realized through adopting precursor A and precursor B. For precursor A, the one-stage tubular furnace with a fixed length was used to prepare the pre-oxidized fiber. The continuous heating mode was applied, and the temperature settings were 200, 215, 238, 255, 260, and 265 °C, respectively. The pre-oxidation time at each temperature was 8, 8, 16, 16, 16, and 8 min. By controlling the relative content of ambient oxygen, the pre-oxidized fibers with different oxygen contents were prepared, as shown in Table 1.

For precursor B, the six-stage continuous furnace was used to prepare the pre-oxidized fiber. The air was adopted as the heat treatment atmosphere. The pre-oxidation time in each furnace was 8, 8, 16, 16, 16, and 8 min, respectively. By adjusting the furnace temperature of each section, the pre-oxidized fibers marked as 1# to 5# were obtained, as shown in Table 2.

### 2.2. Optical Microscope Measurements

**Figure 2:**
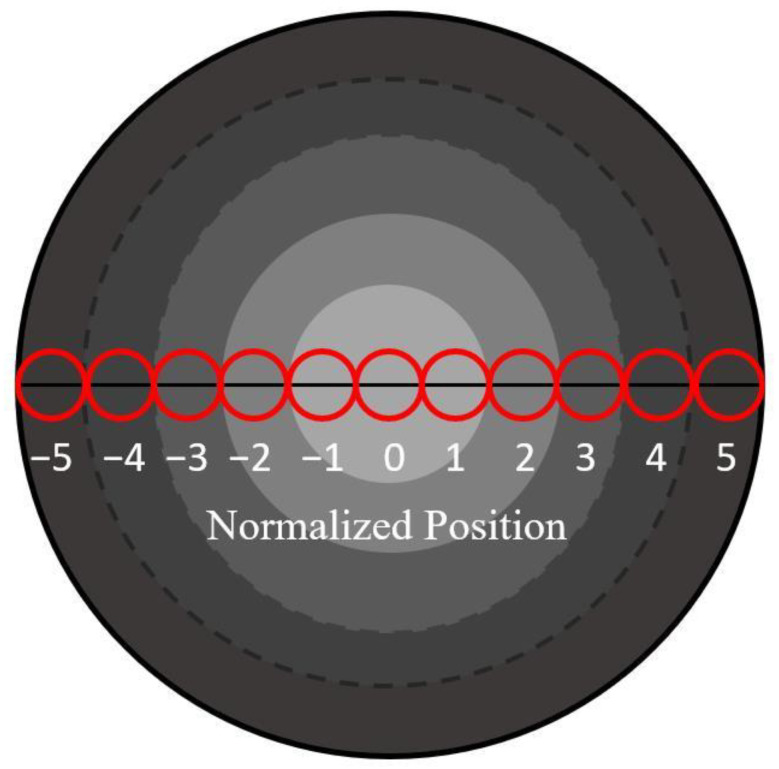
Schematic of radial optical density test of pre-oxidized fiber. (Note: the diameter of each filament for pre-oxidized fiber treated by precursor B was about 11 μm.)

PAN pre-oxidized fiber was embedded into epoxy resin and cured at 75 °C for 24 h. Samples with a thickness of 500 nm were prepared using an EMUC ultrathin slicer. The translucency of the cross-section of the pre-oxidized fibers was tested using a high-power optical microscope (Olympus BX51, Tokyo, Japan). Image Pro Plus software 7.0 was used to analyze the optical density (OD) of the microscopic images, which represented the degree of light transmission of pre-oxidized fibers [21,22]. The magnification of the microscope was 40× with a light intensity of 700 Lx. On the cross-section of the pre-oxidized fiber, taking the center of the circle as the center, the equal circle areas test region with the symmetrical and close arrangement were selected, as shown in Figure 2. The diameter of each circle was 1 μm. Each sample was tested five times, and the mean values were calculated.

### 2.3. C-NMR Measurements

The ^13^C-NMR spectra of PAN pre-oxidized fibers were obtained through a Bruker AV-300 NMR spectrometer, equipped with a 4 mm cross-polarization/magic-angle spinning (CP/MAS) probe, with a rotation speed of 12 kHz. A resonance frequency was 73.5 MHz, the cumulative scanning times were 300–3000 s, and the single scanning time was 5 s. Tetramethylsilane (TMS) was used as the internal reference to determine the chemical shifts.

Relative cyclization index (*R_Cl_*) [23], dehydrogenation index (*G_h_*) [23], and relative content of –C=O functional groups (*R*_–*C*=*O*_) [24] of pre-oxidized fiber were calculated by the following Equations (1)–(3), respectively.

(1) $$R_{Cl}=\frac{I_{C=N}}{I_{CN}+I_{C=N}}\times 100\%$$

(2) $$G_{h}=\frac{I_{C=C}+I_{C=CH}}{I_{CH/CH_{2}}}\times 100\%$$

(3) $$R_{-C=O}=\frac{I_{-C=O}}{I_{CN}+I_{C=N}}\times 100\%$$

where *I* denote the relative intensity, and its subscripts represent the characteristic functional groups.

### 2.4. TGA Measurements

The thermal stability of the pre-oxidized fiber was tested using a Q600 thermogravimetric analyzer (TA, American) in a nitrogen atmosphere with the flow rate 100 mL/min. The samples were heated to 95 °C first at a heating rate of 15 °C/min and held for 5 min, then heated to 800 °C at a heating rate of 15 °C/min. The sample mass was around 4.5 mg. It should be noted that only one sample was adopted in the test. This was because the data of weight variation at elevated temperature during the tests were relatively steady and there was little difference for the samples under the same condition. A similar situation has also been reported by other research work [25,26].

## 3. Results and Discussion

### 3.1. Characteristics of Skin-Core Structure and its Chemical Evolution of PAN Based Pre-Oxidized Fiber Based on Fine Denier Model

**Figure 3:**
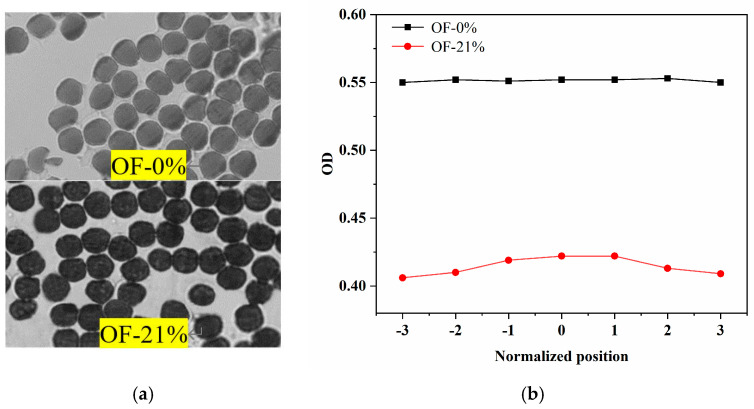
The translucency of the cross-section of the fine denier, PAN pre-oxidized fiber by optical microscope of (**a**) cross-sectional images (the diameter of each filament for pre-oxidized fiber was about 7 µm) and (**b**) optical density.

Precursor fiber A (fine denier) was used for the pre-oxidation treatment in nitrogen and air atmosphere to obtain the pre-oxidized fibers, and they were marked as OF-0% and OF-21%. The cross-sectional images of two pre-oxidized fibers were shown in Figure 3a. The color of OF-0% was lighter, while the color of OF-21% was significantly darker. Figure 3b showed that the optical density value of OF-21% was significantly lower than that of OF-0%, and it increased slightly with the distance close to the core.

Some researchers proposed that the color change of pre-oxidized fiber was related to the oxidation reaction [27]. It was considered that, for the sample of OF-21%, the ambient oxygen had fully diffused into the fiber, which showed the optical density value of its core region after the oxidation reaction was significantly lower than that of the OF-0% sample. Conversely, for the sample of OF-21%, the optical density value of the core region was slightly lower than that of the skin region, which reflected the hindrance of the radial structure on the diffusion of ambient oxygen into the fiber. In fact, there was little difference in the optical density value of the skin/core region for the sample of OF-21%. It could be considered that the degree of oxidation reaction in the fiber radial direction was relatively uniform. Therefore, OF-21% could be considered as the skin structure of the pre-oxidized fiber. For the sample of OF-0%, no oxygen participated in the reaction and no characteristic structure was generated. It could be considered as the core structure in which oxygen did not diffuse into during the pre-oxidation process.

**Figure 4:**
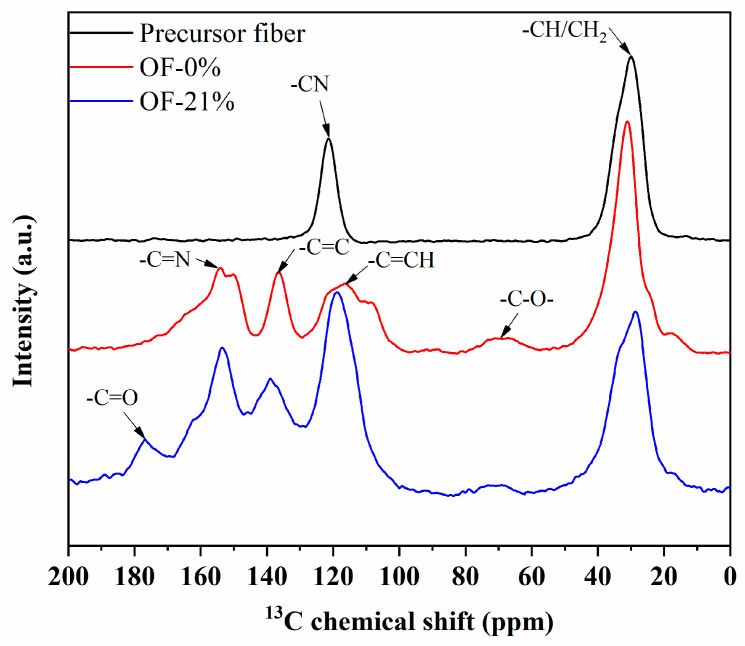
The ^13^C-NMR characterization of the chemical structure of pre-oxidized fibers.

The ^13^C-NMR was used to characterize the chemical structure of OF-0% and OF-21%, which was applied to reveal the difference in skin-core chemical structure of PAN pre-oxidized fibers. As shown in Figure 4, compared with the PAN precursor fiber, the relative content of –C≡N group of pre-oxidized fiber decreased significantly, and the relative content of –C=N, –C=C, and –C=CH group increased. This revealed that the cyclization and dehydrogenation reaction occurred during the pre-oxidation process. The relative content of –C–O– (about 60–80 ppm) increased slightly, which revealed that itaconic acid in the comonomer participated in the pre-oxidation reaction. Owing to the oxidation reaction participated by ambient oxygen, OF-21% corresponding to skin structure produced a new –C=O functional group.

**Figure 5:**
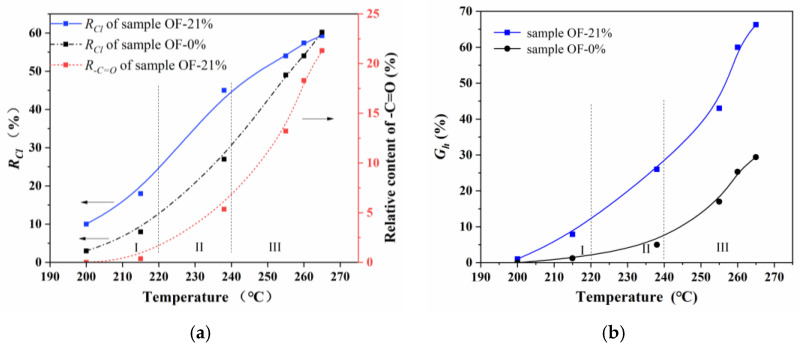
The ^13^C-NMR characterization of chemical structure evolution of pre-oxidized fibers of (**a**) *R_Cl_* and (**b**) *G_h_*.

Based on the ^13^C-NMR curves in Figure 4, the characteristic peaks of each functional group were fitted separately to obtain the relative cyclization rate (*R_Cl_*), dehydrogenation index (*G_h_*), and the relative content of –C=O group (*R_–C=O_*) for the samples of OF-0% and OF-21%. Furthermore, during the pre-oxidation process, the heat-treated samples were selected for the ^13^C-NMR characterization. The above three typical parameters with the increase of heat treatment temperature could be obtained, as shown in Figure 5, which further revealed the chemical evolution characteristics of the skin-core structure in the pre-oxidation process of PAN fibers.

**Figure 6:**
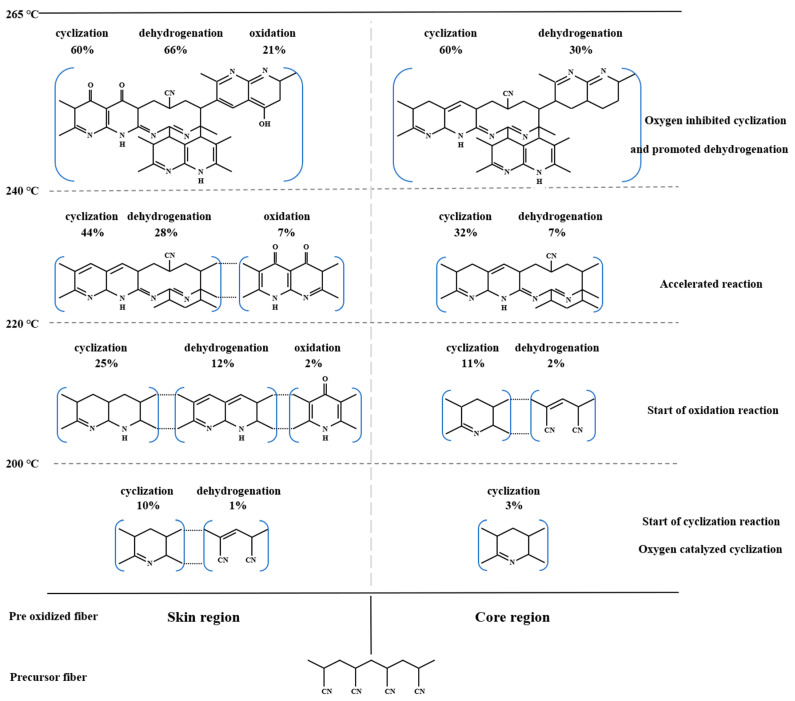
Schematic of the chemical evolution of the skin-core structure of PAN-based pre-oxidized fiber.

As shown in Figure 5, the chemical evolution of a skin-core structure in the pre-oxidation process of PAN fibers with the temperature could be roughly divided into three stages. The first was the initial stage of pre-oxidation reaction (stage I: 200–220 °C). At the same temperature, the relative cyclization degree and dehydrogenation index of OF-21% were significantly higher than that of OF-0%. In addition, the relative content of –C=O groups for OF-21% at this stage was almost zero. The results showed that the oxidation reaction was not apparent in the initial stage. Ambient oxygen mainly prompted cyclization and dehydrogenation at the lower temperature. Furthermore, the relative contents of cyclization and dehydrogenation structure in the skin region of PAN fiber were significantly higher than that of the core region. The second was the middle stage of the pre-oxidation reaction (stage II: 220–240 °C). At the same temperature, the degree of cyclization and dehydrogenation of OF-21% was significantly higher than that of OF-0%. With the increase of heat treatment temperature, the relative content of –C=O for OF-21% increased slowly, while the overall content was relatively low. The results showed that in the middle stage of the pre-oxidation reaction, the oxidation reaction began slowly, and the catalytic cyclization of oxygen continued. This further promoted the relative content of cyclization and dehydrogenation structure in the skin region compared to the core region. The third was the latest stage of pre-oxidation reaction (stage III: >240 °C). At the same temperature, the degree of cyclization and dehydrogenation of OF-21% was higher than that of OF-0%. However, with the increase of heat treatment temperature, the relative content of –C=O for OF-21% increased rapidly, and the difference of cyclization degree between OF-21% and OF-0% decreased gradually. When the heat-treated temperature increased to 265 °C, there was little difference in the cyclization degree. The results showed that in the latest stage, the oxidation reaction intensified and promoted the dehydrogenation reaction, which continued. While the catalytic cyclization of oxygen decreased as the cyclization reaction in the pre-oxidation stage approached the end. Therefore, the oxidation and dehydrogenation structures in the skin region were significantly larger than that of the core. The cyclization degrees of the skin and core region after the pre-oxidation process were similar. The chemical evolution of the skin-core structure of the PAN-based pre-oxidized fiber was shown in Figure 6.

### 3.2. Radial Structure Characteristics of PAN-Based Pre-Oxidized Fiber Affected by Ambient Oxygen Concentration

With the assumption that the oxygen content was gradually decreased along the fiber radial structure during the pre-oxidation process, precursor fiber A (fine denier) was used to pre-oxidize with different oxygen concentrations. The prepared pre-oxidized fibers were denoted as OF-2.5%, OF-5%, OF-10%, and OF-21%. Adding the above-mentioned OF-0%, the chemical structures of different radial positions for PAN fiber from core to skin could be characterized.

**Figure 7:**
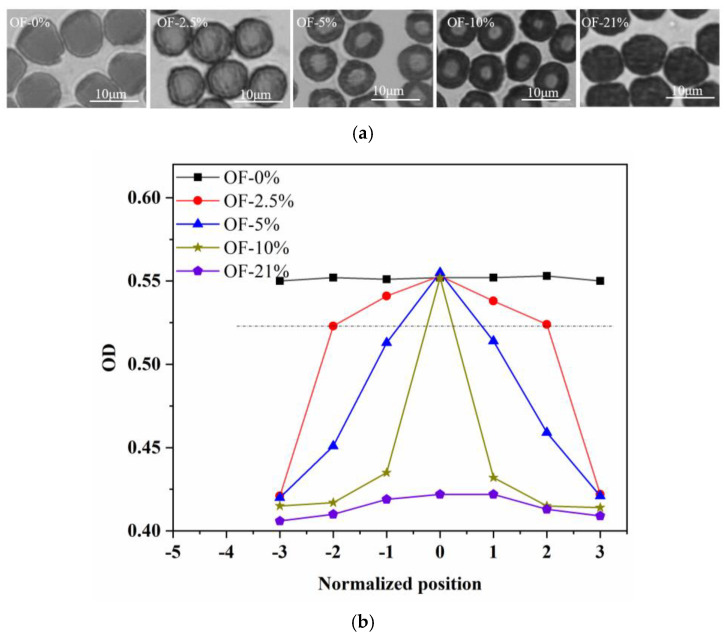
The translucency of the cross-section of the fine denier PAN pre-oxidized fiber by optical microscope of (**a**) cross-sectional transmittance images (Note, the diameter of each filament for pre-oxidized fiber was about 7 µm) and (**b**) optical density.

The cross-sectional transmittance images of the above five pre-oxidized fibers were shown in Figure 7. Compared with the OF-0% (core structure), the oxygen permeability of the PAN fiber was enhanced with the increase of ambient oxygen content. The area of the core region became gradually smaller, and the area of the skin region became gradually larger. The radial structure showed the gradient change. When the ambient oxygen content reached 21%, the oxygen entered into the center of the fiber, which resulted in the more uniform oxidation reaction degree in the fiber radial direction. the optical densities of the core and skin were almost the same.

**Figure 8:**
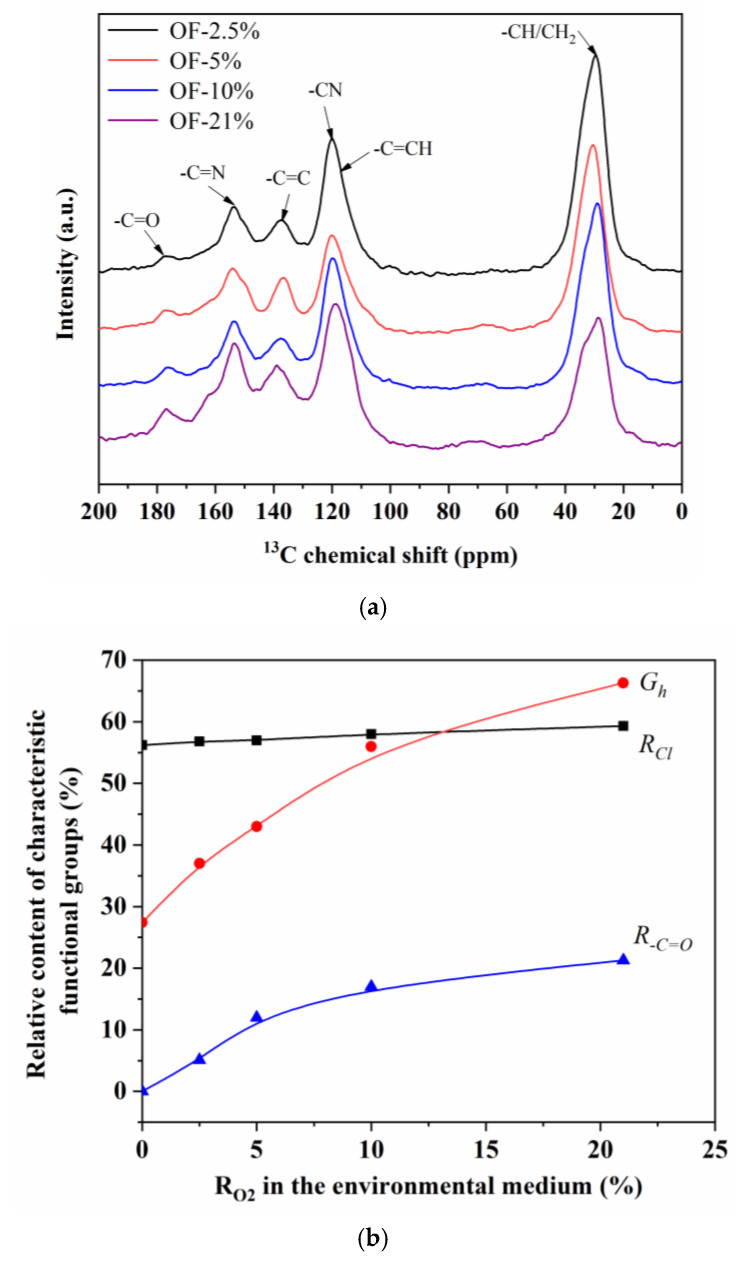
The ^13^C-NMR characterization of chemical structure evolution of pre-oxidized fibers. (**a**) the ^13^C-NMR spectrum and (**b**) relative content of characteristic functional groups.

The ^13^C-NMR was used to characterize the differences of the chemical structure for four kinds of pre-oxidized fibers of OF-2.5%, OF-5%, OF-10%, and OF-21%, as shown in Figure 8. It could be concluded that in the oxygen atmosphere, the generated chemical functional groups of four kinds of pre-oxidized fibers had no apparent change, while the relative content was different. As shown in Figure 8b, the characteristic peaks of each functional group were fitted separately to obtain the relative cyclization rate (*R_Cl_*), dehydrogenation index (*G_h_*), and the relative content of –C=O group (*R_–C=O_*) of four kinds of pre-oxidized fibers. As shown, there was little difference in the relative cyclization rate for the four pre-oxidized fibers. However, the degree of dehydrogenation and oxidation reaction increased significantly with the increase of ambient oxygen content, which reflected the real radial structure of the pre-oxidized fiber. The degree of cyclization reaction had little difference from core to skin, while the degree of dehydrogenation and oxidation reaction increased gradually.

### 3.3. Radial Structure of PAN Pre-Oxidized Fiber

Precursor fiber B (conventional size) was used for pre-oxidation treatment under the multi-stage temperature regulation in air, and the pre-oxidized fibers with the number of 1# to 5# were prepared as shown in Table 2. Compared with 1#, the pre-oxidation temperature of 2# was reduced in three stages; 3# was increased in three stages; 4# was treated by increasing temperature in stage III; and 5# was treated by increasing temperature in stages I and II.

**Figure 9:**
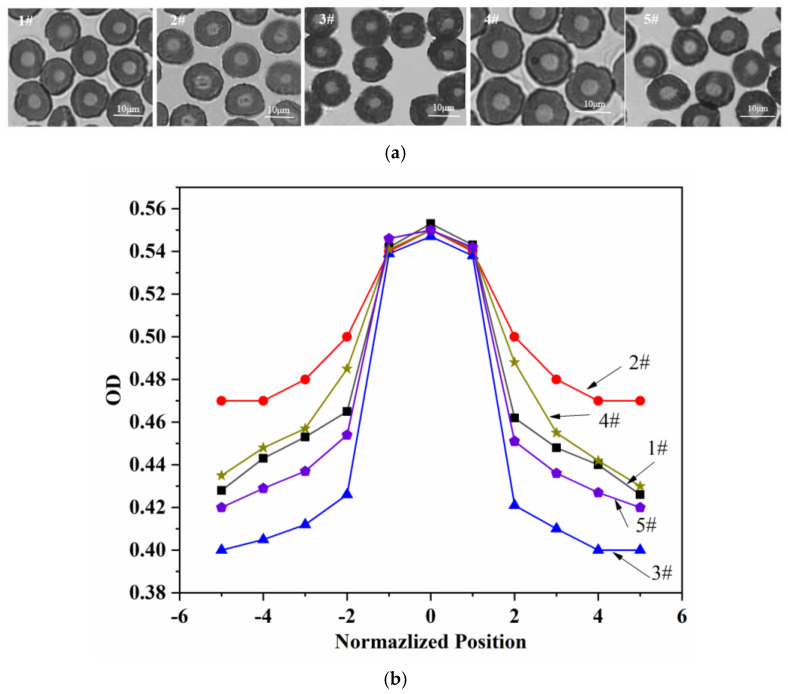
The translucency of the cross-section of the fine denier PAN pre-oxidized fiber by optical microscope of (**a**) cross-sectional transmittance images (Note, the diameter of each filament for pre-oxidized fiber was about 11 µm) and (**b**) optical density.

The cross-sectional transmittance images of five kinds of pre-oxidized fibers were shown in Figure 9a. It could be observed that five fibers had skin-core structures. The optical density results in Figure 9b showed that the core regions of five fibers were basically the same, which reflected the dependence of the core region on the fiber size and on the ambient oxygen concentration. When the fiber size and the ambient oxygen concentration were constant, there was little difference in the degree of oxygen diffusion into the fibers. In addition, the optical density values of five fibers in the skin were different under the effect of heat treatment temperature. Peebles et al. [28] proposed that the chromogenic group of pre-oxidized fiber was about the conjugated chromogenic structures and contained –C=N and –C=O. It was considered that the difference of optical density values in the skins revealed the difference of pre-oxidation structure related to heating temperature. Combined with the chemical structural evolution during the pre-oxidization process of the skin-core structure of PAN fibers in Figure 6, it was considered that compared with 1#, the cyclization, dehydrogenation, and oxidation reactions of 2# were reduced due to the overall reduction of heat temperature. As a result, the conjugated structure was reduced. At the same radial position of fiber, the optical density of the skin was relatively larger. The degree of cyclization, dehydrogenation, and oxidation reaction of 3# increased by increasing the heating temperature and the conjugated structure increased, which made the optical density of the skin at the same radial position relatively small. Next 5# was prepared by increasing the temperature in stages I and II. At this stage, the cyclization reaction could be promoted by higher temperature. Some researchers proposed that the cyclization reaction was the basis of oxidation reaction during the pre-oxidation process. The oxidation reaction based on enough cyclization reaction could promote to form more conjugated structures in the system [29,30]. So, the relative optical density of skin at the same radial position decreased. The 4# sample was prepared by increasing temperature in stage III. At this stage, the degree of oxidation reaction was significantly improved. However, the degree of cyclization reaction in stages I and II were not enhanced. It affected that the formed conjugate structure was imperfect compared with 5#. Correspondingly, the optical density of the skin at the same radial position was relatively increased.

**Figure 10:**
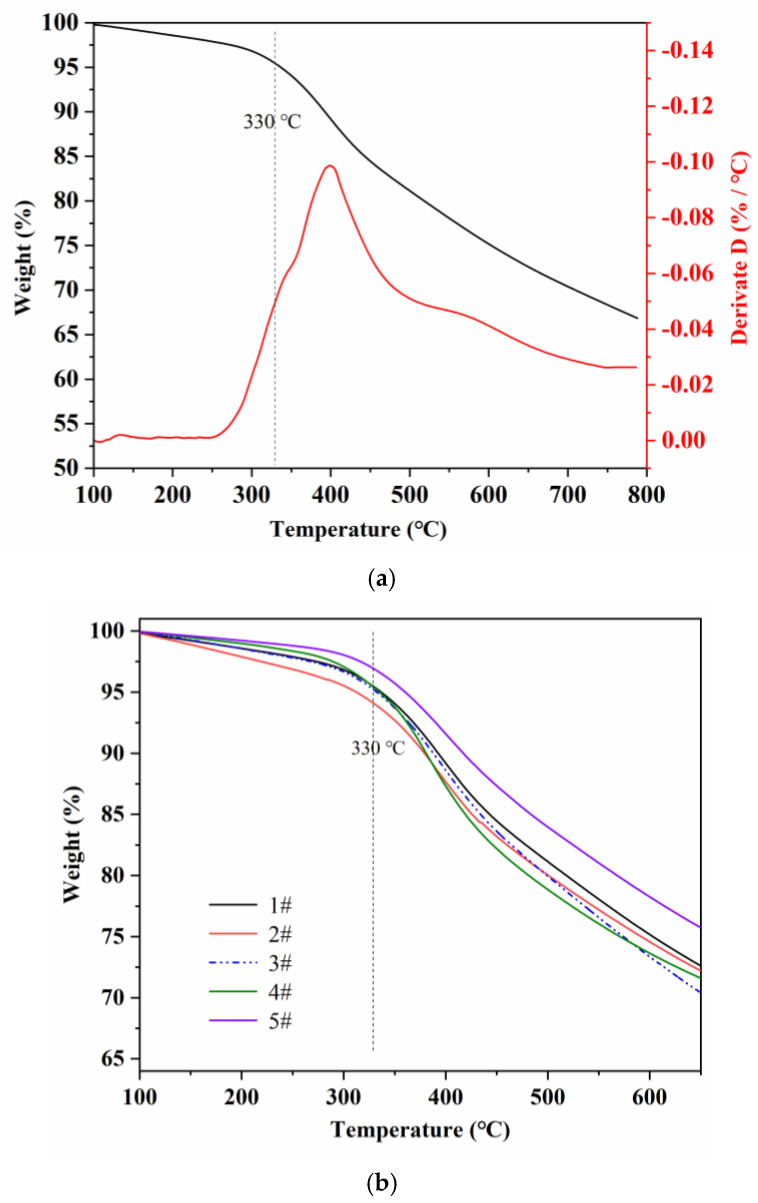
Thermogravimetric curve of 1# to 5# samples (**a**) TGA/DTGA curve of 1# pre-oxidized fiber (**b**) TGA curve of 1# to 5# samples.

Figure 10 showed the thermogravimetric curves of five kinds of pre-oxidized fibers in a nitrogen atmosphere, which further revealed the stable structure of these fibers formed during the pre-oxidation process. As shown in Figure 10a, the DTG curve showed that the thermal weight loss of pre-oxidized fiber was mainly divided into two stages. In the first stage (about 100–330 °C), the weight loss rate was about 5%. In the second stage (330–650 °C), the weight loss rate was about 25%. Researchers [31] proposed that the first stage of weight loss was the pyrolysis product of the non-cyclized and cross-linked part of the pre-oxygenated fiber, while the second stage of weight loss was the small molecule by-products in the process of forming large conjugate structure through the thermal crosslinking and thermal condensation. Figure 10b showed that compared with 1#, the thermal weight loss of 3# was relatively maximum and that of 5# was relatively minimum. It indicated that the ladder structure of 5# generated after the pre-oxidation was relatively more in amount.

**Figure 11:**
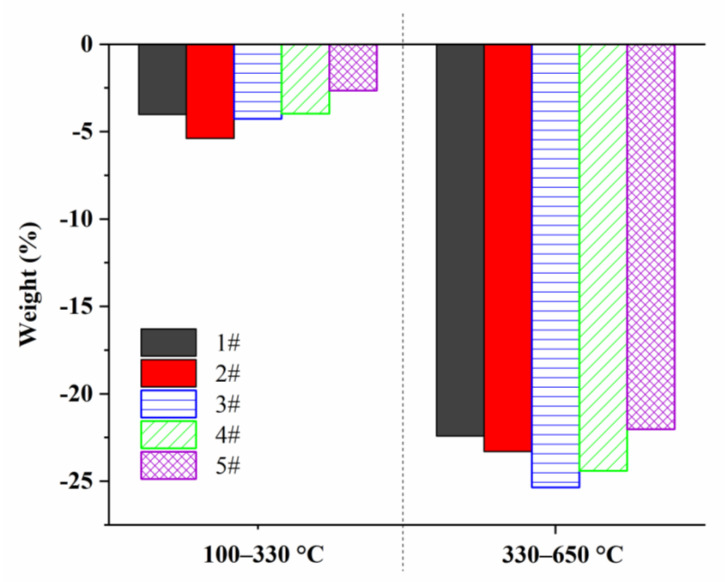
Thermal weight loss rate in the first and second stages for 1#–5# pre-oxidized fibers.

Furthermore, the differences in weight loss rate of five samples in the first and second stages were shown in Figure 11. Among them, compared with 1# sample, the weight loss rate of 2# sample was higher in the first and second stages, and the weight loss rate of 5# sample was the lowest in both stages. While the weight loss rates of 3# and 4# samples were similar to that of 1# samples in the first stage, which increased significantly in the second stage.

Combined with the different formation structure of the above 1# to 5# pre-oxidized fiber, it was considered that compared with 1#, the degree of cyclization, dehydrogenation, and oxidation reaction of 2# was relatively lower. In the first stage of weight loss, since 2# sample had more non-cyclized and crosslinked parts, it would produce more pyrolysis products after the further rapid heating. In the second stage of weight loss, the smaller trapezoidal structure was thermally crosslinked and condensed into the larger conjugated structure. Due to the imperfect structure of cyclization, dehydrogenation, and oxidation in the first stage, it was difficult to form the larger conjugated structure in the follow-up. Many imperfect small trapezoidal structures were cracked and removed in this process, which affected the thermal weight loss rate of 2# sample, which was greater than that of 1# in the second stage. The cyclization, dehydrogenation, and oxidation structures of 3# were improved. While the dehydrogenation and oxidation reaction structure of 4# were relatively more. The weight loss rate of the two samples in the first stage had changed little compared with 1#. However, too much oxidation structure would be removed in the form of small molecules at the higher temperature (above 350 °C). It caused the cracking of a large number of oxygen-containing trapezoidal structures. Thus, the thermal weight loss rate of both samples in the second stage was significantly higher than 1#. Compared with 3# samples, 5# was treated by further increasing the stage I temperature. It promoted the formation of more cyclization reaction structures. Then suitable dehydrogenation and oxidation structures were established on the basic structure. The weight loss rate of 5# was the lowest in the whole thermogravimetric process. The results showed that by using the pre-oxidation temperature control method of 5#, it meant that moderately increasing the temperature in the early and middle stages of the pre-oxidation process could promote the formation of more cyclized structures and more stable cross-linked ladder structures in the skin of fiber. It could comprehensively improve the thermal stability of pre-oxidized fibers.

## 4. Conclusions

In this paper, a series of fine denier model fibers were prepared by adjusting the oxygen concentration to describe the structural characteristics at different radial regions of pre-oxidized fibers. The evolution mechanism of the radial structure of pre-oxidized fiber, with the increase of heat treatment temperature, was studied by the microstructure analysis. The following conclusions can be drawn:

1. The pre-oxidation reaction of PAN fiber resulted in the existence of a skin-core structure. Along the fiber radial direction from skin to core, the degree of cyclization changed little, and the dehydrogenation and oxygen-containing structure gradually decreased. Specifically, the oxygen-containing functional groups in the core decreased to the lowest level or even disappeared.
2. With the increase of a heat treatment temperature, a three-stage variation of the skin-core structure of the PAN fiber was observed. For stage I, there were mainly cyclization and dehydrogenation structures in the skin region. For stage II, the oxygen-containing structure in the skin increased slowly. Lastly for stage III, the cyclization structure in the skin and core was similar.
3. Upon increasing the heat treatment temperature in stage III, the cyclization reaction in the skin was inhibited, and there were relatively more dehydrogenation and oxidation reaction structures. Moderate increasing the heat treatment temperature in stages I and II could promote the formation of a more cyclized structure and a more stable cross-linked ladder structure in the skin of fiber.

## Figures and Tables

**Table 1:** Prepared pre-oxidized fiber with different oxygen content, marked as OF.

Sample No.	Relative Content of Oxygen
OF-0%	0%
OF-2.5%	2.5%
OF-5%	5%
OF-10%	10%
OF-21%	21%

Note: OF-0% was treated in nitrogen atmosphere; OF-2.5% was treated in nitrogen atmosphere containing 2.5% oxygen; OF-5% was treated in nitrogen atmosphere containing 5% oxygen; OF-10% was treated in nitrogen atmosphere containing 10% oxygen; and OF-21% was treated in air.

**Table 2:** Pre-oxidized fiber prepared at different heat treatment temperatures.

Sample No.	Furnace Temperature of Each Section (°C)					
	1	2	3	4	5	6
1#	200	215	238	255	260	265
2#	200	213	235	250	255	260
3#	200	220	243	255	265	270
4#	200	215	243	258	260	265
5#	210	225	238	255	260	265

## Data Availability

Not applicable.
